# Analysis of temporal correlation in heart rate variability through maximum entropy principle in a minimal pairwise glassy model

**DOI:** 10.1038/s41598-020-72183-4

**Published:** 2020-09-18

**Authors:** Elena Agliari, Francesco Alemanno, Adriano Barra, Orazio Antonio Barra, Alberto Fachechi, Lorenzo Franceschi Vento, Luciano Moretti

**Affiliations:** 1 Dipartimento di Matematica “Guido Castelnuovo”, Sapienza Università di Roma, P. le A. Moro, 00185 Roma, Italy; 2 Dipartimento di Matematica e Fisica “Ennio De Giorgi”, Università del Salento, Via per Arnesano, 73100 Lecce, Italy; 3 Institute of Nanotechnology, National Research Council (CNR-NANOTEC), Campus Ecotekne, Via Monteroni, 73100 Lecce, Italy; 4 Istituto Nazionale di Fisica Nucleare (INFN), Campus Ecotekne, Via Monteroni, 73100 Lecce, Italy; 5 Department of Environmental Engineering, University of Calabria (INICAL-DIAM), 87035 Cosenza, Italy; 6 Politecnico Internazionale “Scientia et Ars” (POLISA), Largo Intendenza, 89900 Vibo, Valentia Italy; 7 Department of Cardiology “C. & G. Mazzoni”, Hospital (APH), Via degli Iris, 63100 Ascoli-Piceno, Italy

**Keywords:** Computational biophysics, Cardiovascular diseases, Complex networks, Statistical physics, Biomarkers

## Abstract

In this work we apply statistical mechanics tools to infer cardiac pathologies over a sample of *M* patients whose heart rate variability has been recorded via 24 h Holter device and that are divided in different classes according to their clinical status (providing a repository of labelled data). Considering the set of inter-beat interval sequences $\{\mathbf {r}(i) \} = \{ r_1(i), r_2(i), \ldots , \}$, with $i=1,\ldots ,M$, we estimate their probability distribution $P(\mathbf {r})$ exploiting the maximum entropy principle. By setting constraints on the first and on the second moment we obtain an effective pairwise $(r_n,r_m)$ model, whose parameters are shown to depend on the clinical status of the patient. In order to check this framework, we generate synthetic data from our model and we show that their distribution is in excellent agreement with the one obtained from experimental data. Further, our model can be related to a one-dimensional spin-glass with quenched long-range couplings decaying with the spin–spin distance as a power-law. This allows us to speculate that the 1/*f* noise typical of heart-rate variability may stem from the interplay between the parasympathetic and orthosympathetic systems.

## Introduction

**Figure 1:**
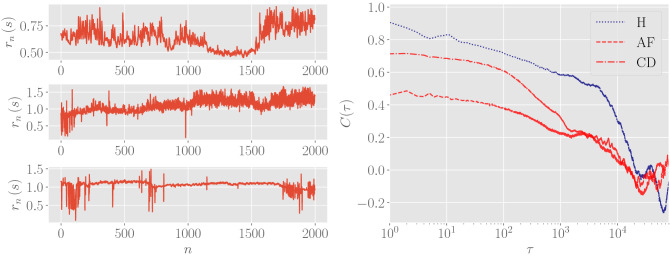
Left: examples of the bare RR time series for a single patient for each class; the window depicted is restricted to the first 2000 beats. Right: examples of autocorrelation functions for a single patient for each class. The dotted blue line refers to a healthy patients, while red are patients with AF (dashed curve) and CD (dash-dotted line). Notice that, in any case, the autocorrelation remains positive over several orders of magnitude and this is a consequence of the scale-free behavior of the series power spectral density (see also Fig. 7, upper panel).

Heart-rate variability (HRV) analysis constitutes a major tool for investigating the mechanisms underlying the complex and chaotic cardiac dynamics as well as for identifying general features discriminating the clinical status of patients^1,11,14,15,16,18,19^. To this aim a fundamental observable is the RR series $\mathbf {r} = \{r_1, r_2, \ldots \}$, where $r_n$ is the temporal distance between the *n*-th and the $(n+1)$-th R peaks in a ECG recording (see Fig. 1 left panel). Several approaches have been carried out in the past in order to address the HRV analysis from this observale (see e.g.,^17,37,38^). For instance, in^1,2^ the problem of classification of heart failures via time-series analysis is translated into a search for clusterization in a high-dimensional space.

Interestingly, the intrinsic variability in heat rate ultimately stems from the interplay of the sympathetic and the parasympathetic nervous system. In this work, exploiting statistical inference approaches, we aim to unveil any signature of this underlying autonomic neural regulation. To this scope we will study HRV in the temporal and in the frequency domain, and at different levels of aggregation (in the higher one the sample is made of all available data, in the lower one we build different sub-samples pertaining to patients displaying a different clinical status: healthy, suffering from cardiac decompensation, suffering from atrial fibrillation).

The statistical inference approach we adapt to the present case of study is the *maximum entropy framework*^3^ recently developed and applied to pitch sequences for capturing melodic styles^4^. In general, the maximum entropy principle has been widely applied to biological problems, ranging from the cellular scales of neuronal^6^ and immunological settings^5^, up to social scales of bird’s flocks^8^ and ant’s communications^9^. By this technique, we search for the minimal-structure probabilistic model compatible to our data; more precisely, we consider the family of probability distributions $P(\mathbf {r})$ over the sequences of inter-beat intervals $\mathbf {r}$ whose lowest momenta match the empirical ones and, among all the elements of this family we select the one corresponding to the maximum entropy. As a direct consequence of the definition of entropy in terms of the logarithm of the probability distribution $P(\mathbf {r})$ over the inter-beat sequences $\mathbf {r}$, this approach returns an exponential family $P(\mathbf {r}) \sim e^{-H(\mathbf {r})}$, where $H(\mathbf {r})$ can be interpreted as a cost function (or Hamiltonian in a physical jargon). By requiring a match on the first two moments only (i.e., by requiring that the theoretical average and two-point correlation provided by the model are quantitatively consistent with the empirical ones), $H(\mathbf {r})$ results in a pairwise ($r_n ,r_m$) cost-function, as standard in Physics.

Of course, recovering the complex structure hidden in RR series by a relatively simple pairwise model has several advantages: on the one hand, the low number of parameters prevents from over-fitting, on the other hand, the inferred cost-function can be framed in a statistical mechanics context (see e.g.,^5–9^) and we can therefore rely on several powerful techniques and on a robust Literature. In particular, we will show that, despite its simplicity, such a pair-wise model is able to capture the complex nature of the temporal correlation between beats which emerges experimentally; in fact, the coupling between two beat-intervals $r_n$ and $r_{n+\tau }$ turns out to be long-range (i.e., displays a power-law decay with the distance $\tau$) and frustrated (i.e., the couplings between two beats can be positive and negative). In a statistical-mechanical jargon, this system is referred to as a two-body spin-glass with power-law quenched interactions.

Remarkably, frustration in couplings, which is a key feature of spin-glasses, means the existence of competitive driving forces and it is natural to look at this emerging feature in our model as the hallmark of the interplay between the parasympathetic and orthosympathetic systems (indeed, while the first one tends to increase the distance between RR peaks, i.e., to lower the heart rate^18^, the latter tends to decrease it^20^). We speculate that these competing interactions may be responsible for the well-known 1/*f* noise shown by HRV^12,13,21^: spin-glasses typically display a chaotic dynamics^22–24^ spread over several timescales^25^ and their power spectrum density is power-law^32–34^. In fact, here we show that the autocorrelation in the $\{ r_n \}$ series decays in the beat number as $n^{-1}$ and its related power spectrum decays in frequecy as $f^{-1}$. Incidentally, we notice that variables whose fluctuations display 1/*f* noise are widespread, ranging from inorganic (e.g., condensed^26^, granular^27^, etc.) to organic (e.g. in DNA sequences^28^, membrane channels^29^) matter and, even broadly, in Nature (e.g. ranging from earthquakes^35^ to off-equilibrium flows of current trough resistors^36^, to the whole self-organized criticality^30,31^).

## Results

### Summary of experimental data

In this Section, we give some details about the data and the quantities considered in our analysis. This research has been accomplished as a part of the Project *MATCH* (Mathematical Advanced Tools to Catch Heart-Rate-Variability), a scientific cooperation among Ascoli Piceno Hospital (APH), POLISA, University of Calabria and University of Salento: Holter recordings have been acquired at APH and their data-base built at POLISA during the period 2016–2019.

The database is made of ECG recordings on $M=348$ patients, wearing an Holter device for nominal 24 h. From these recordings we extract the RR series

1 $$\begin{aligned} \{ \mathbf {r}(i) \}_{i=1,\ldots ,M} = \{ r_n (i)\}_{\begin{array}{c} i=1,\ldots ,M \\ n=1,\ldots ,N_i ,\end{array}} \end{aligned}$$

where *i* labels the patient and *n* labels the number of beats in each sequence (which is order of $10^5$ and depends on the patient). Patients belong to three classes, according to their clinical status: healthy individuals (H), individuals with atrial fibrillation (AF) and individuals with congestive heart failure (hereafter simplified as *cardiac decompensation*) (CD). Their number is $M_H = 149$, $M_{AF}=139$, and $M_{CD} =60$, respectively; of course, $M=M_H + M_{AF}+M_{CD}$. In Fig. 1 (left) we show examples of the series $\mathbf {r}(i)$ for three patients belonging to the different classes.

In order to make a meaningful comparison of the variability among the RR series $\mathbf {r} (i)$ of different patients, we standardize them with respect to their temporal mean and standard deviation, so that the study of HRV is recast in the study of fluctuations of the standardized RR series around the null-value. More precisely, we introduce

2 $$\begin{aligned} z_n(i) = \frac{r_n(i) - \langle {\mathbf {r}}(i) \rangle }{\text {std} [\mathbf {r}(i)] }, \,\, \text {for} \,\, n=1, \ldots , N \end{aligned}$$

or, in vectorial notation,

3 $$\begin{aligned} \mathbf {z}(i) = \frac{\mathbf {r}(i) - \langle \mathbf {r}(i) \rangle }{\text {std} [\mathbf {r}(i)] }, \end{aligned}$$

where we defined

4 $$\begin{aligned} \langle \mathbf {r} (i) \rangle = \frac{1}{N_i} \sum _{n=1}^{N_i} r_n (i), \quad \langle \mathbf {r}^2(i) \rangle = \frac{1}{N_i} \sum _{n=1}^{N_i} r^2_n (i), \quad \text {std}[\mathbf {r}(i)] = \sqrt{\langle \mathbf {r}^2(i) \rangle - \langle \mathbf {r} (i) \rangle ^2}. \end{aligned}$$

**Figure 2:**
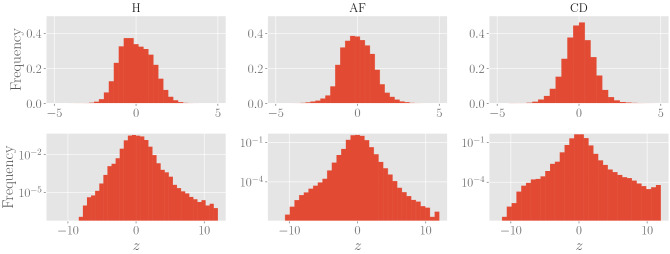
Histograms of the standardized values $\{ \mathbf {z}(i) \}$ divided by classes: left panels are build by collecting data from healthy patients, middle panels are build by collecting data from patients suffering from atrial fibrillation and right panels are build by collecting data from patients suffering from cardiac decompensation. In the first row, we reported relative frequencies in the natural scale, while the second row we reported relative frequencies in the logarithmic scale.

The raw histograms for the standardized inter-beat intervals in the three classes of patients are shown in Fig. 2: notice that the frequency distributions exhibit heavy-tails.

We consider the points in the standardized RR series as random variables sampled by a hidden stochastic process, in such a way that the value of $z_n(i)$ at a given step *n* depends in principle on all the values $\{z_m(i)\}_{m<n}$ taken in the previous steps $m<n$ since the beginning of sampling. From this perspective, a meaningful observable to look at is the auto-correlation function at a distance $\tau$, defined as

5 $$\begin{aligned} C(i, \tau ) = \frac{1}{N_i - \tau } \sum _{n=1}^{N_i -\tau } \big ( z_n(i) -\langle {z}(i) \rangle _{+}\big )\big (z_{n+\tau }(i)- \langle {z}(i)\rangle _{-}\big ), \end{aligned}$$

where $\langle {z}(i) \rangle _{+} = \frac{1}{N_i-\tau } \sum _{n=1}^{N_i-\tau } z_n (i)$ and $\langle {z}(i)\rangle _{-} = \frac{1}{N_i-\tau } \sum _{n=\tau }^{N_i} z_n (i)$. Given the standardization over the whole segment $[1, N_i]$, as long as $\tau \ll N_i$, we expect that $\langle {z}(i)\rangle _{+}$ and $\langle {z}(i)\rangle _{-}$ are both close to zero and shall be neglected in the following (indeed, we checked that this is the case, since $\langle {z}(i)\rangle _{+},\langle {z}(i)\rangle _{-} \sim 10^{-15}\div 10^{-17}$). Then, the auto-correlation function we measure simply reduces to

6 $$\begin{aligned} C(i, \tau ) = \frac{1}{N_i - \tau } \sum _{n=1}^{N_i - \tau } z_n(i) z_{n+\tau }(i) . \end{aligned}$$

Some examples of the autocorrelation function for patients of the three classes are reported in the right plot of Fig. 1, where we stress that the autocorrelation is non-null over a large $\tau$ window and its shape is patient-dependent. The last property stems from the 1/*f* noise shown by HRV which, in turns, yields to dominant contributions in the low-frequency domain of the RR series.

Finally, we introduce a further average operation, this time on the sample of patients, namely, we define

7 $$\begin{aligned} \mathbb {E}_{class}(\mathbf {z})= & {} \frac{1}{M_{class}} \sum _{i \in class} \mathbf {z} (i), \end{aligned}$$

8 $$\begin{aligned} \mathbb {E}_{class}(\mathbf {z^2})= & {} \frac{1}{M_{class}} \sum _{i \in class} \mathbf {z}^2 (i),\end{aligned}$$

9 $$\begin{aligned} \mathbb {E}_{class}(C(\tau ))= & {} \frac{1}{M_{class}} \sum _{i \in class}C(i, \tau ) . \end{aligned}$$

where $class \in \{ H, AF, CD\}$ and with “$i \in class$” we mean all the indices corresponding to patients belonging to a certain class. In the following we will consider the vectors $\mathbf {z}$ as random variables sampled from an unknown probability distribution $P^{true}_{class}(\mathbf {z})$, which we will estimate by the probability distribution $P_{class}(\mathbf {z})$ characterized by a minimal structure and such that its first and second moments are quantitatively comparable with $\mathbb {E}_{class}(\mathbf {r})$, $\mathbb {E}_{class}(\mathbf {z^2})$ and $\mathbb {E}_{class}(C(\tau ))$, respectively.

### Discussion on the model and on the inferential procedure

Our atomic variable is the sequence $\{ z_1, z_2, \ldots , z_N \}$ and, as anticipated above, we denote with $P(\mathbf {z})$ the related probability distribution emerging from the inferential operations on the sample of experimental data. The Shannon entropy $\widetilde{H}[P(\mathbf {z})]$ associated to $P(\mathbf {z})$ is

10 $$\begin{aligned} \widetilde{H}[P(\mathbf {z})] = - \int {\mathrm {d} \mathbf {z} P(\mathbf {z}) \ln P(\mathbf {z})}. \end{aligned}$$

According to the maximum entropy principle, we look for the distribution $P(\mathbf {z})$ that maximizes $\widetilde{H}[P(\mathbf {z})]$ and such that its moments match those evaluated experimentally, in particular, here the we choose to apply the constraints on the one-point and two-points correlation function that is, $\mathbb {E}_{class}(\mathbf {z})$, $\mathbb {E}_{class}(\mathbf {z^2})$ and $\mathbb {E}_{class}(C(\tau ))$, respectively. To lighten the notation hereafter these moments shall be referred to simply as, respectively, $\mu ^{(1)}$, $\mu ^{(2)}$ and $C(\tau )$, without specifying the class. In fact, the inferential procedure works analogously regardless of the class, the latter affecting only the quantitative value of the parameters occurring in $P(\mathbf {z})$. Constraints are set via Lagrange multipliers $(\lambda _0, \lambda _1, \lambda _2,\lambda _\tau )$ in such a way that the problem is recast in the maximization of the functional

11 $$\begin{aligned} \widetilde{H}_{\lambda _0,\lambda _1,\lambda _2,\lambda _\tau }[P(\mathbf {z})]&=\widetilde{H}[P(\mathbf {z})]+ \lambda _0\left( \int {\mathrm {d} \mathbf {z} \, P(\mathbf {z})}-1\right) \nonumber \\&\quad + \lambda _1\left( \sum _{n=1}^{N}\int {\mathrm {d} \mathbf {z} \, P(\mathbf {z}) z_n }-N\mu ^{(1)}\right) + \lambda _2\left( \sum _{n=1}^{N}\int {\mathrm {d} \mathbf {z} \, P(\mathbf {z}) {z}_n^2}-N\mu ^{(2)}\right) \nonumber \\&\quad + \sum _{\tau =1}^{N}\lambda _\tau \left( \sum _{n=1}^{N-\tau }\int {\mathrm {d} \mathbf {z} \, P(\mathbf {z}) {z}_n z_{n+\tau }}-(N-\tau )C(\tau )\right) , \end{aligned}$$

where integration is made over $\mathbb {R}^N$. Note that, while the derivation with respect to $\lambda _1$, $\lambda _2$ and $\lambda _\tau$ ensure, respectively, the agreement between the theory and the experiments at the two lowest orders, i.e. the temporal average $\mu ^{(1)}$, the second moment $\mu ^{(2)}$ and the auto-correlation function $C(\tau )$, $\lambda _0$ guarantees that $P(\mathbf {z})$ is normalized, so that $P(\mathbf {z})$ is a probability distribution function. In the asymptotic limit of long sampling ($N \rightarrow \infty$) and under a stationarity hypothesis (see^4^ for a similar treatment and the section dedicated to Methods for the proof), the solution of the extremization procedure, returning the probability of observing a certain sequence $\mathbf {z}$, is given by

12 $$\begin{aligned} P(\{z_n\}_{n=1}^\infty )=\frac{1}{Z} \left( \prod _{n=1}^{\infty } P_0 (z_n)\right) \exp \left( \sum _{n=1}^\infty \sum _{\tau =1}^\infty J(\tau )z_n z_{n+\tau }+h\sum _{n=1}^\infty z_n\right) , \end{aligned}$$

where *h* and $J(\tau )$ can be estimated from available data (*vide infra*). Here, $P_0$ is the $\mathscr {N}(0,1)$ distribution and plays the role of prior for the variable $z_n$, the parameter $J(\tau )$ represents the pairwise interaction between elements at non-zero distance $\tau$ in the series (notice that each element occurs to be coupled to any other), and the parameter *h* represents the bias possibly affecting the single value in the sequence (and it is expected to be zero as we standardized the RR series). The factor *Z* plays here as a normalization constant, like the partition function in the statistical mechanics setting^10^. Notice that the interaction between two elements $r_n$ and $r_m$ ($n>m$) depends on the distance $\tau =n-m$, but not on the particular couple considered. This stems from a“stationary hypothesis”, meaning that one-point and two-point correlation functions calculated on a segment spanning $O(\tau \ll N)$ elements along the series are approximately the same and since the starting time of sampling is arbitrary, we get that $J(n,m) = J(m-n)$.

The standard inference setup for the model parameters is based on a *Maximum (log-)Likelihood Estimation* (MLE), i.e. the maximization of the function

13 $$\begin{aligned} \mathscr {L}(\mathbf {J}, h \vert \mathscr {D})= -\frac{1}{M} \sum _{\mathbf {z} \in \mathscr {D}} \log P(\mathbf {z}\vert \mathbf {J},h), \end{aligned}$$

where $\mathscr {D}$ is the time-series database (of a given class) and where we made clear the dependence of *P* on the model parameters. However, such an approach requires the computation of the whole partition function *Z*, which is numerically hard in this case. Then, we chose to adopt as objective function for the inference procedure the *pseudo-(log-)likelihood* function^4^:

14 $$\begin{aligned} \mathscr {L}(\mathbf {J}, h \vert \mathscr {D})=-\frac{1}{M} \sum _{\mathbf {z} \in \mathscr {D}}\log P(z_{L+1}\vert \{z_n\}_{n=1}^L), \end{aligned}$$

that is, given *L* observations in a fixed time-series $\mathbf {z}$, we maximize the conditional probability to observe the value $z_{L+1}$ at the successive time step (i.e., we are introducing a cut-off in the interaction range). Further, we make two main modifications with respect to the standard pseudo-likelihood approach: (i) in order to use the entire available time-series in our database, we also adopt a window average procedure; (ii) we add regularization terms in order to prevent divergence for the model parameters. A detailed discussion is reported in Appendix 4.2. Our objective function is therefore given by

15 $$\begin{aligned} \mathscr {L}^{(\text {reg})} (\mathbf {J}, h \vert \mathscr {D})=\frac{1}{M} \sum _{\mathbf {z} \in \mathscr {D}}\left[ -\frac{1}{2(N-T)}\sum _{n=T}^{N-1}\big (z_{n+1}-h-\sum _{\tau =1}^{T}J(\tau ) z_{n+1-\tau } \big )^2-\frac{\lambda }{2}h^2-\frac{\lambda }{2}\sum _{\tau =1}^T f(\tau ) J(\tau )^2\right] , \end{aligned}$$

where *T* is the largest $\tau$ we want to consider (namely *T* must be larger that the maximal decorrelation time), $\lambda$ is the regularization weight and $f(\tau )$ is a temporal regularizer that prevents the elements of $J(\tau )$ to get too large for large $\tau$ (see Appendix 4.2 for a detailed description).

**Figure 3:**
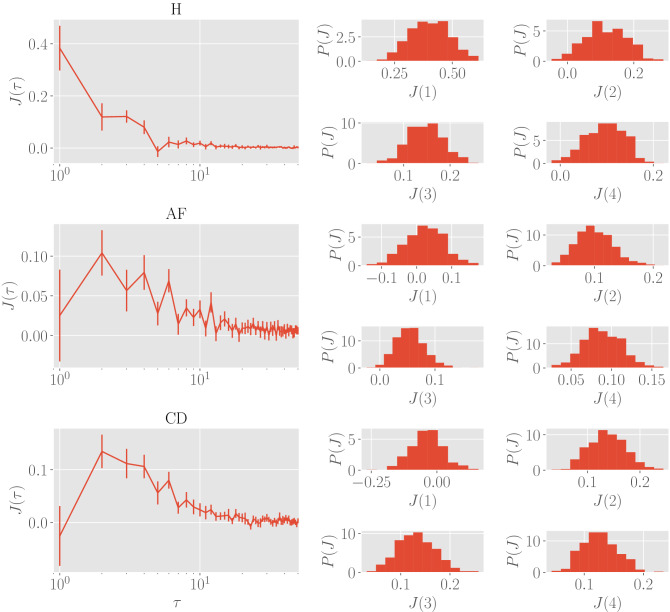
Inference results for delayed interactions. Left column: the plots show the results of the inference procedure (distinguishing between the clinical status) for the first 50 $\tau$s. Right: frequency distribution of the *J*s for some selected values of $\tau$ (i.e. $\tau =1,2,3,4$). In both cases,the statistics consists in $M=500$ different realizations of the $J(\tau )$ which are realized by randomly extracting different mini-batches, each with size $n=20$. We stress that some frequency distributions present tails on negative values of *J* for some $\tau$. This means that frustrated interactions are also allowed, implying that the system is fundamentally complex, i.e. a *glassy hearth*.

The inference method allows us to determine the values of the parameters $J(\tau )$ and *h* as well as their uncertainties $\sigma _{J(\tau )}$ and $\sigma _h$. As for the parameter *h*, due to series standardization, its value, evaluated over the different classes, is expected to be vanishing (this is indeed the case as it turns out to be $h\sim 10^{-3}$ with a related uncertainty of the same order). As for the pairwise couplings, we find that for all the classes considered, $J(\tau )$ is significantly non-zero only for relatively small values of $\tau$, with a cutoff at $T \sim 10^2$, and, for a given $\tau < T$, the coupling does not display a definite sign, that is, for pairs $(z_n, z_{n+\tau })$ and $(z_m, z_{m+\tau })$ at the same distance $\tau$ the related couplings can be of opposite signs. These results are shown in Fig. 3: in the left column we reported the inferred $J(\tau )$ with the associated uncertainties for all $\tau$, and in each panel in the right we reported the frequency distributions for the first values of $\tau$ as examples.

In order to study how decorrelation of RR intervals takes place, it is interesting to study how the interaction vector $J(\tau )$ (regardless of its sign) vanishes as the delay time $\tau$ increases. We found that the long delay time behavior of the magnitude (i.e. disregarding the signs and oscillatory characteristics) of the interactions is well-described by a power law of the form

16 $$\begin{aligned} |J(\tau )|_\text {leading}\sim {A}\cdot \tau ^{-\beta }. \end{aligned}$$

**Figure 4:**
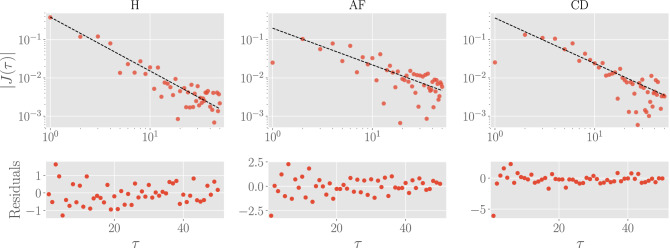
Leading behavior of magnitude of the delayed interactions. In the upper panel, we reported the absolute value of the delayed interactions $J(\tau )$ and the relative best fit. In the lower panel, we reported the residuals (normalized by the uncertainty at each point $\tau$) of the experimental data with respect to the best fit function. We stress that, even if the interactions $J(\tau )$ are far from the fitting curve (in the log-log scale, see first row), they are compatible within the associated uncertainties, as remarked by the residual plots.

We thus fitted the tails of the inferred $J(\tau )$ with this trial function (the range of fitted points is chosen in order to maximize the adjusted $R^2$ score). In Table 1, we report best-fit parameters, the adjusted $R^2$ score and the reduced $\chi ^2$. It is interesting to note that the scaling parameter $\beta$ is around 1, meaning that, for each of the three classes, the leading behavior of the interactions at large $\tau$ is $\sim 1/\tau$ (as we will see later, the same scaling also characterize the power spectral density in the Fourier domain, see Fig. 7). In the upper row of Fig. 4, we depicted with red circles the results of the inference procedure (once taken their absolute values), while the best fit of the general trend is represented with dashed black lines. In the lower row of the same figure, we also reported the residuals of the experimental values with respect to the best fit (normalized to the corresponding uncertainty). Apart from the first few points in the AF and CD cases, we see that the residuals are distributed in a range of at most $2 \sigma _{J(\tau )}$ (where $\sigma _{J(\tau )}$ is the standard deviation at each $\tau$ point), and, in particular, for sufficiently long $\tau$ (where oscillations are softened), experimental values are always contained in the range $[-\sigma _{J(\tau )},\sigma _{J(\tau )}]$, implying that the leading behavior of the delayed interaction is well-captured by $\sim 1/\tau$ noise both in our datasets as well as in the model’s prediction.

**Table 1:** Best fit values regarding the scaling reported in Eq. (16). For each class, we report the best-fit parameters *A* and $\beta$, as well as the adjusted $R^2$ and the reduced $\chi ^2$ scores quantifying the fit goodness.

Class	*A*	$\beta$	$\bar{R}^2$	$\chi ^2/\text {DOF}$
H	$0.38 \pm 0.04$	$1.41\pm 0.04$	0.75	0.33
AF	$0.20 \pm 0.03$	$0.96\pm 0.05$	0.79	0.42
CD	$0.37 \pm 0.05$	$1.2 \pm 0.05$	0.78	0.46

To summarize, here we highlight the main results.

- Couplings can be both positive and negative (see Fig. 3), defining the heart as a complex glassy system.
- The coupling magnitude decays in $\tau$ as a power-law whose leading order is $\sim 1/\tau$ (see Fig. 4).
- The coupling magnitude displays a sharp scaling $1/\tau$ solely in healthy patients, while for the remaining patients it display a *bump* in the short time-scales (see Fig. 3 and the residual plots in Fig. 4).
- By fitting data via Eq. (16), we obtain estimates for the power-law exponent $\beta$ (as reported in Table 1): interestingly, different classes are associated to different best-fit values of $\beta$, in such a way that classification of cardiac failures via HRV via this route seems possible.

### Discussion on the model and on the generalization procedure

Once the model and the related parameters are inferred for each of the three classes, we can use the original sequences $\{ \mathbf {z} \}$ to generate synthetic sequences $\{\tilde{\mathbf{z}}\}$ of length $N-T$. The procedure followed to get the synthetic sequence is briefly described hereafter, while an extensive explanation is provided in App. 4.3.

For any class, we consider our estimate for $J(\tau )$, along with the estimate $\sigma _{J(\tau )}$ of its uncertainty, and we build the noisy estimate for $J(\tau )$, that is $\bar{J}(\tau )=J(\tau ) + \delta J(\tau )$ where $\delta J(\tau )= \eta \sigma _{J(\tau )}$ and $\eta$ is a $\mathscr {N}(0,1)$ random variable. Next, taken a certain $\{ \mathbf {z} \}$, we convolve it with $\bar{J}(\tau )$ and this returns $\{\tilde{\mathbf{z}}\}$. Of course, due to the initial standardization of the RR series, the inference procedure returned a vanishing bias parameter *h*, hence the synthetic series will also be centered at zero. However, a synthetic sequence is no longer standardized and this is done by hand.

**Figure 5:**
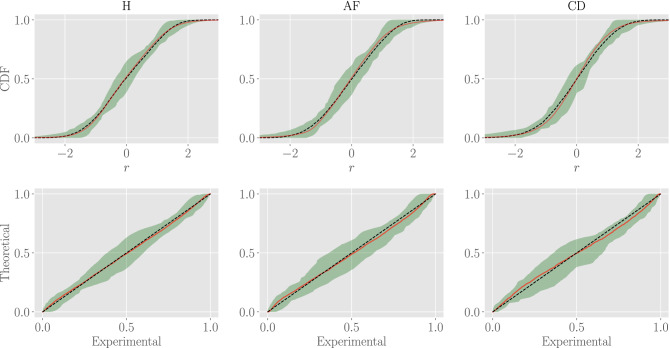
Comparison between posterior distributions for experimental and synthetic data. First row: comparisons between the empirical cumulative distributions for both experimental (solid red lines) and resampled (black dashed lines) populations for all of the three classes. Second row: probability plots for the two populations of data (i.e. empirical *versus* theoretical ones, red solid lines) for all of the three classes. The black solid curves are the identity lines for reference. The green region is the confidence interval with $p=0.95$.

Then, it is natural to compare the synthetic sequences and the experimental ones. We generate a sample of data with the same size of the experimental data available, and we compute the empirical cumulative distribution function for both the experimental and synthetic data in order to compare them: results are reported in Fig. 5. In the first row, we directly compare the experimental (red solid line) and the synthetic (black dashed line) cumulative distributions highlighting an excellent agreement for all the classes. This is then corroborated by checking the probability plots in the same figure (second row): here, the red solid line shows the synthetic cumulative distribution versus experimental cumulative distribution, while the black dashed curve is the identity line. The green regions in the plot are confidence intervals with $p=0.95$.

**Figure 6:**
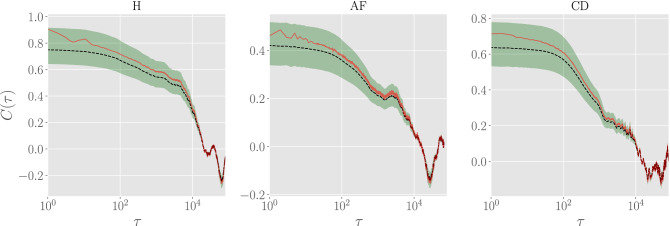
Comparison between autocorrelation functions for experimental and synthetic data. The autocorrelation function for one patient randomly extracted from the experimental data-set (red solid lines) is compared with the median autocorrelation function obtained from the synthetic dataset (black dashed lines). Notice that the former falls in the confidence interval with $p=0.68$ (green region) of the latter.

Next, we test whether the model is able to effectively capture correlation in the RR series, in particular by comparing experimental auto-correlation functions and their predicted counterparts. However, since autocorrelation functions are individual-dependent, starting from a single (randomly chosen) RR series we generate 100 synthetic series with different realizations of the $\bar{J}(\tau )$ according to the above mentioned procedure (see also App. 4.3). In this way, we can use our estimation of the uncertainties on $J(\tau )$ in order to give a confidence interval for our predictions. In Fig. 6 we compare the auto-correlation functions for the experimental series and for the synthetic series; for the latter we also highlight the confidence interval with $p=0.68$. More precisely, we depict the experimental (red solid line) and theoretical (black dashed line) auto-correlation functions and see that the former always fall inside the confidence interval of the re-sampled series (the green region). Thus, we can conclude that our inferred minimal pairwise model is able to effectively capture the temporal autocorrelation in the RR series.

**Figure 7:**
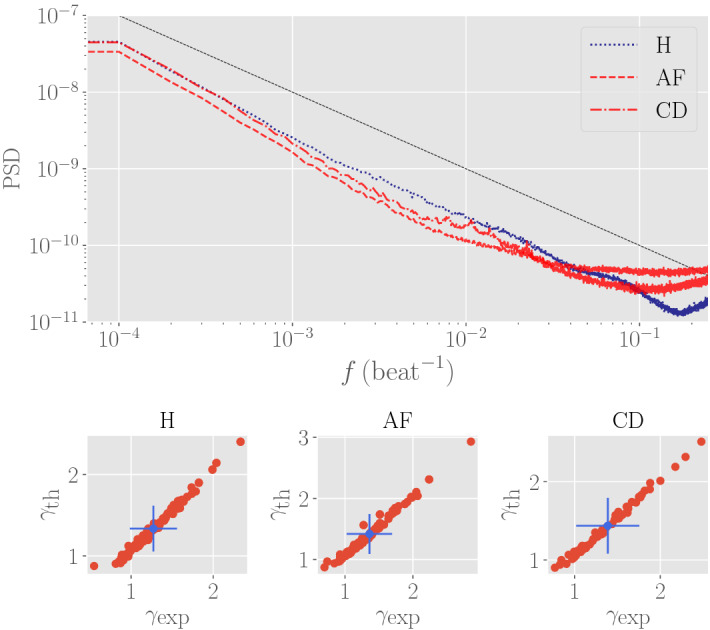
Top: empirical power spectral density (PSD). The dotted blue line refers to a healthy patients, while the red ones refer to patients with AF (dashed curve) and CD (dash-dotted line). The PSD is computed according to the Welch procedure with $50\%$ windows overlap. The black continuous curve is the expected 1/*f*-noise distribution for visual comparison. Bottom: scatter plot for the scaling exponent of the PSD (in the region $10^{-4}$ e $10^{-2}$ Hz); in particular, we take the simple average over the synthetic realizations, the red spots are the exponent for the single patient (notice that the uncertainties over the synthetic realization are much smaller and are not visible in the plot), and the blue spot marks the average over all patients (both experimental and synthetic) with the relative uncertainties.

As a final comment, we also looked at the power spectrum density (PSD) of the provided datasets $\{\mathbf {z}\}$ that, as expected (see e.g.,^12,13,21^), displays the long tail 1/*f* (see Fig. (7), upper panel) and we made the following comparison: for all the patients, we evaluated its PSD and in the region $[10^{-4}, 10^{-2}]$ Heartbeat$^{-1}$ we fit with a power-law

$$\begin{aligned} \text {PSD}(f) = \alpha \cdot f^{-\gamma } \end{aligned}$$

where $\gamma \sim 1$ and its value is taken as the *x*-coordinate of that patient in the lower panels of Fig. (7). The corresponding *y*-value is obtained by calculating the PSD over 100 synthetic RR-series generated by convolution with the empirical series playing as seed and using as value of $\varvec{J}$ the one pertaining to the class the patient belongs to (H, AF, CD); results are in good agreement on the diagonal.

## Conclusion

Several past studies have highlighted that heart-rate fluctuations, in healthy individuals, exhibit the characteristic 1/*f* *noise* (see^12^ and references therein). Deviations from this behavior can in fact be associated to cardiac pathologies such as atrial fibrillation or congestive heart failure^21^. In this work we tried to deepen the mechanisms possibly underlying this peculiar behavior, both in healthy and in compromised subjects.

To this aim we exploited inferential tools derived from statistical mechanics (i.e. the maximum entropy principle) to construct a probability distribution $P(\mathbf {r})$ characterizing the occurrence of a RR series $\mathbf {r}$. By requiring that $P(\mathbf {r})$ is minimally structured (i.e., prescribing the maximum entropy) and that $P(\mathbf {r})$ correctly matches the empirical first and second moments, we end up with a probabilistic model analogous to a spin-glass where quenched couplings $J(\tau )$ among spins exhibit frustration and a power-law decay with the distance $\tau$ between spin pairs. This kind of system is known to display chaotic dynamics spread over several timescales and the 1/*f* noise. We thus speculate that the presence of competitive driving force are key features for the emergence of the rich phenomenology displayed by heart-rate and we are naturally tempted to identify the two opposite driving forces with the sympathetic and parasympathetic systems.

We stress that our model for $P(\mathbf {r})$ is robustly checked and that tests performed over different classes of patients (diplaying a different clinical status) allows us to candidate the exponent $\beta$ controlling the coupling decay $J(\tau ) \sim \tau ^{-\beta }$ as an indicator for classify the patient clinical status.

## Methods

This Section is devoted to the description of the statistical model and the optimization rules used in our inference procedure.

### The two-body statistical mechanical model from maximum entropy principle

As stated in Sec. 2, the probabilistic model we use to frame the analysis of heart-rate variability contained in the standardized RR series $\{z_n\}_{n=1}^N$ emerges as the solution of extremization procedure of the constrained Shannon entropy functional

17 $$\begin{aligned} \widetilde{H}_{\lambda _0,\lambda _1,\lambda _2,J}[P(\mathbf {z})]&=\widetilde{H}[P(\mathbf {z})]+ \lambda _0\left( \int {\mathrm {d} \mathbf {z} \, P(\mathbf {z})}-1\right) \nonumber \\&\quad + \lambda _1\left( \sum _{n=1}^{N}\int {\mathrm {d} \mathbf {z} \, P(\mathbf {z}) z_n }-N\mu ^{(1)}\right) + \lambda _2\left( \sum _{n=1}^{N}\int {\mathrm {d} \mathbf {z} \, P(\mathbf {z}) {z}_n^2}-N\mu ^{(2)}\right) \nonumber \\&\quad + \sum _{\tau =1}^{N}\lambda _\tau \left( \sum _{n=1}^{N-\tau }\int {\mathrm {d} \mathbf {z} \, P(\mathbf {z}) {z}_n z_{n+\tau }}-(N-\tau )C(\tau )\right) . \end{aligned}$$

Here, we recall that the first term is the standard Shannon entropy for probability distribution for continuous variables, i.e.

18 $$\begin{aligned} \widetilde{H}[P(\mathbf {z})]= -\int \mathrm {d} \mathbf {z} P(\mathbf {z})\log P (\mathbf {z}), \end{aligned}$$

while the other terms are constraints with Lagrangian multipliers $\lambda _0$, $\lambda _1$, $\lambda _2$ and $\lambda _\tau$. The extremization with respect to these parameters leads to the following conditions:

19 $$\begin{aligned} \int {\mathrm {d} \mathbf {z} \, P(\mathbf {z})}=1,\quad \frac{1}{N}\sum _{n=1}^{N}\int {\mathrm {d} \mathbf {z} \, P(\mathbf {z}) z_n }=\mu ^{(1)},\quad \frac{1}{N} \sum _{n=1}^{N}\int {\mathrm {d} \mathbf {z} \, P(\mathbf {z}) {z}_n^2}=\mu ^{(2)},\quad \frac{1}{N-\tau }\sum _{n=1}^{N-\tau }\int {\mathrm {d} \mathbf {z} \, P(\mathbf {z}) {z}_n z_{n+\tau }}=C(\tau ), \end{aligned}$$

i.e. that the function $P (\mathbf {z})$ is a probability distribution and that the moments up to the second order are captured by the experimental temporal average $\mu ^{(1)}$, the temporal standard deviation $\mu ^{(2)}$ and the auto-correlation function $C(\tau )$. The extremization with respect to the function *P* leads to the explicit form of the solution, i.e.

20 $$\begin{aligned} \log P(\mathbf {z})=\lambda _0-1+\lambda _1\sum _{n=1}^{N}z_n+\lambda _2 \sum _{n=1} ^N z_n^2+ \sum _{\tau =1}^{N}\lambda _\tau \sum _{n=1}^{N-\tau } {z}_n z_{n+\tau }, \end{aligned}$$

which can be rewritten as

21 $$\begin{aligned} P(\mathbf {z})=\text {cost}\cdot \exp \left( \lambda _1\sum _{n=1}^N z_n+\lambda _2\sum _{n=1}^N z_n^2+\sum _{\tau =1}^N\sum _{n=1}^{N-\tau } \lambda _\tau z_n z_{n+\tau }\right) . \end{aligned}$$

The constant in the latter equation is computed by using the normalization property of the probability distribution $P(\mathbf {z})$, and it is given by

22 $$\begin{aligned} \text {cost}^{-1}=Z=\int \mathrm {d} \mathbf {z} \exp \left( \lambda _1\sum _{n=1}^N z_n+\lambda _2\sum _{n=1}^N z_n^2+\sum _{\tau =1}^N\sum _{n=1}^{N-\tau } \lambda _\tau z_n z_{n+\tau }\right) , \end{aligned}$$

where we used the letter *Z* to make contact with the notion of partition function from the Thermodynamics dictionary. Since the model is essentially Gaussian, we can directly compute the partition function (at least, in formal way) as

23 $$\begin{aligned} Z=\int \mathrm {d} \mathbf {z} \exp \left( \lambda _1 \mathbf {E} ^T \cdot \mathbf {z}+\mathbf {z}^T (\lambda _2 \mathbb I+{\pmb \lambda })\mathbf {z}\right) = (-\pi )^{N/2} \text {det}^{-1/2}(\lambda _2 \mathbb I +{\pmb \lambda })\exp (-\lambda _1^2\mathbf {E}^T(\lambda _2\mathbb I+{\pmb \lambda })^{-1}\mathbf {E}). \end{aligned}$$

where $\mathbf {E}=(1,1,\dots ,1)$ is a *N*-dimensional vector of ones and we defined the interaction matrix $({\pmb \lambda })_{n,m}=\sum _{\tau =1}^N\delta _{n,m-\tau }\lambda _\tau$ (which turns out to be an upper triangular Toeplitz matrix with zeros on the main diagonal). Because of this, the determinant of the kernel $\lambda _2 \mathbb I + {\pmb \lambda }$ is trivially $\text {det} (\lambda _2 \mathbb I+{\pmb \lambda })=\lambda _2^{N}$. We can now determine the relation between the temporal average and standard deviation in terms of the model parameters. These relations read as

24 $$\begin{aligned} \mu ^{(1)}= & {} \langle \frac{1}{N} \sum _{n=1}^N z_n\rangle _{\text {th}}= \frac{1}{N}\frac{\partial \log Z}{\partial \lambda _1}=-\frac{2}{N}\lambda _1 \mathbf {E}^T(\lambda _2\mathbb I+{\pmb \lambda })^{-1}\mathbf {E}, \end{aligned}$$

25 $$\begin{aligned} \mu ^{(2)}= & {} \langle \frac{1}{N} \sum _{n=1}^N z^2_n \rangle _{\text {th}} =\frac{1}{N}\frac{\partial \log Z}{\partial \lambda _2}=-\frac{1}{2 \lambda _2}-\frac{\lambda _1^2}{N}\frac{\partial }{\partial \lambda _2}\mathbf {E}^T(\lambda _2\mathbb I+{\pmb \lambda })^{-1}\mathbf {E}, \end{aligned}$$

where $\langle \cdot \rangle _{\text {th}}$ is the theoretical average as defined in Eq. 19.

Since $\mathbf {z}$ is temporally standardized, we directly get $\lambda _1=0$ and $\lambda _2=-1/2$. However, we left the former as a free parameter to be inferred and check *a posteriori* that it is consistent with zero. In order to get contact with Physics’ dictionary, we rename the Lagrangian multipliers $\lambda _1=h$ and $\lambda _\tau =J(\tau )$, playing the role of external magnetic field and two-body interactions respectively. Then, the solution of the maximum entropy problem (after some rearrangements of the sum indices) is given by

26 $$\begin{aligned} P(\mathbf {z})=\frac{1}{Z} \left( \prod _{n=1}^{N} P_0 (z_n)\right) \exp \left( \sum _{n=1}^{N-1}\sum _{\tau =1}^{N-n} J(\tau )z_n z_{n+\tau }+h\sum _{n=1}^N z_n\right) , \end{aligned}$$

where $P_0(z)$ is the Gaussian distribution $\mathscr {N}(0,1)$ and *Z* is the partition function.

### The inference setup

The determination of the model parameters $J(\tau )$ and *h* is based on a maximum likelihood approach. As stated in the model description, the usage of the full probability is computationally untractable (because of the high dimensionality of the integral in the partition function),thus we use a maximum pseudo-likelihood approach^4^ (namely a tractable asymptotic correct estimator of the likelihood), in which the fundamental object to be maximized is the conditional probability that, given the observations $\{z_n\}_{n=1}^L$, the successive observation is equal to experimental data $z_{L+1}$:

27 $$\begin{aligned} P\big (z=z_{L+1}\vert \{z_n\}_{n=1}^L\big )=\frac{P\big (\{z_n\}_{n=1}^{L+1}\big )}{P\big (\{z_n\}_{n=1}^L\big )}=\frac{Z^{(L)}}{Z^{(L+1)}}\frac{\big (\prod _{n=1}^{L+1} P_0 (z_n)\big )\exp \Big (\sum _{n=1}^{L}\sum _{\tau =1}^{L+1-n} J(\tau )z_n z_{n+\tau }+h\sum _{n=1}^{L+1} z_n\Big )}{\big (\prod _{n=1}^{L} P_0 (z_n)\big )\exp \Big (\sum _{n=1}^{L-1}\sum _{\tau =1}^{L-n} J(\tau )z_n z_{n+\tau }+h\sum _{n=1}^L z_n\Big )}. \end{aligned}$$

The second factor is easy to handle with, while the partition function can be evaluated by using the fact that $\int dz P\big (z\vert \{z_n\}_{n=1}^L\big )=1$, so that we finally have

28 $$\begin{aligned} P\big (z=z_{L+1}\vert \{z_n\}_{n=1}^L\big )=\frac{\exp \Big (\log P_0 (z_{L+1})+\sum _{\tau =1}^{L} J(\tau )z_{L+1-\tau }z_{L+1} +h\, z_{L+1}\Big )}{\int dz \exp \Big (\log P_0 (z)+\sum _{\tau =1}^{L} J(\tau ) z_{L+1-\tau }z+h\, z\Big )}. \end{aligned}$$

Notice that eqs. 27–28 are, in fact, approximations given that interactions between past (before $L+1$) and future (after $L+1$) events are neglected. Now, since the prior is Gaussian, we can directly integrate the denominator for carrying out a closed form for the conditional probability. Thus, we get

29 $$\begin{aligned} P\big (z=z_{L+1}\vert \{z_n\}_{n=1}^L\big )=\frac{1}{\sqrt{2\pi }}\exp \Big (-\frac{1}{2} \big (z_{L+1}-\sum _{\tau =1}^L J(\tau )z_{L+1-\tau }-h\big )^2\Big ). \end{aligned}$$

The MLE is based on the maximization of this conditional probability, or equivalently of the pseudo log-likelihood, which is composed by quantities of the form

30 $$\begin{aligned} \log P(z=z_{L+1}\vert \{z_n\}_{n=1}^L)= -\frac{1}{2}\big (z_{L+1}-\sum _{\tau =1}^L J(\tau )z_{L+1-\tau }-h\big )^2, \end{aligned}$$

where we discarded unessential constant terms. Since we would like to infer the first values of the delayed interaction vector $J(\tau )$ and since the RR time-series have a size which is of the order of $10^5$, it is better to use a sliding-window average approach, whose functioning is ensured by the stationary hypothesis. In this way, we can also perform a temporal average over all a single RR time-series. Supposing we want to infer the first *T* elements of the delayed interaction $J(\tau )$ (i.e. we truncate long-term correlations) and given the time-series $\{z_n\}_{n=1}^N$ of length *N*, we define the individual log-likelihood as

31 $$\begin{aligned} \mathscr {L} (\mathbf {J}, h\vert \{z_n\}_{n=1}^N )=&-\frac{1}{N-T}\sum _{L=T}^{N-1} \log P(z_{L+1}\vert \{z_n\}_{n=L-T+1}^L)\nonumber \\ =&-\frac{1}{2(N-T)}\sum _{L=T}^{N-1}\big (z_{L+1}-h-\sum _{\tau =1}^{T}J(\tau ) z_{L+1-\tau } \big )^2. \end{aligned}$$

In order to prevent the parameters to acquire large values, we also introduce some regularization. For the bias, we simply add a quadratic penalization term: $\mathscr {R}(h)=-\lambda h^2/2$. Concerning the interaction vector, in order to discourage the algorithm to generate spurious correlation for high $\tau$, we introduce a penalization which depends on the delay time $\tau$, i.e.

32 $$\begin{aligned} \mathscr {R}(\mathbf {J})= -\frac{\lambda }{2}\sum _{\tau =1} ^T f(\tau ) J(\tau )^2. \end{aligned}$$

However, in order to ensure not to destroy correlation for interesting values of $\tau$, we adopt a mild regularizer. In all of our tests, we found that a good choice is $f(\tau )=\log ^2(1+\tau )$. Putting all pieces together, we have the regularized individual pseudo log-likelihood

33 $$\begin{aligned} \mathscr {L}^{(\text {reg})} (\mathbf {J}, h\vert \{z_n\}_{n=1}^N )&=-\frac{1}{2(N-T)}\sum _{L=T}^{N-1}\big (z_{L+1}-h-\sum _{\tau =1}^{T}J(\tau ) z_{L+1-\tau } \big )^2\nonumber \\&\quad -\frac{\lambda }{2}h^2-\frac{\lambda }{2}\sum _{\tau =1} ^T f(\tau ) J(\tau )^2. \end{aligned}$$

The whole pseudo-likelihood is the average over the set $\mathscr {D}_c$ time-series in each given class (where $c \in \{ H, AF, CD\}$):

34 $$\begin{aligned} \mathscr {L}^{(\text {reg})} ( \mathbf {J}, h\vert \mathscr {D}_c)=\frac{1}{M_c} \sum _{\mathbf {z}\in \mathscr {D}_c}\mathscr {L}^{(\text {reg})} (\{z_n\}_{n=1}^N\vert \mathbf {J}, h), \end{aligned}$$

where $M_c$ is the number of examples in the class. By adopting a standard gradient descent (GD) approach, we can derive the following optimization rules:

35 $$\begin{aligned} \delta J(\tau )= & {} \sum _{L=T}^{N-1} \Delta _L z_{L+1-\tau }-\lambda f(\tau ) J(\tau ), \end{aligned}$$

36 $$\begin{aligned} \delta h= & {} \sum _{L=T}^{N-1} \Delta _L -\lambda h, \end{aligned}$$

where

37 $$\begin{aligned} \Delta _L=\frac{1}{N-T}(z_{L+1}-h-\sum _{\tau =1}^T J(\tau )z_{L+1-\tau }). \end{aligned}$$

In order to speed up the inference procedure, we use a AdaGrad^39^ adaptation method for the gradient descent rules (35,36). Since we want a uncertainties estimation for the coupling matrix $J(\tau )$, we proceed in the following way: better than to realize a single delayed interaction for the whole database (for each class), we minimize the pseudo log-likelihood to *M* random subsets of cardinality *n* of the database of each class (i.e. the gradients are averaged with respect to this minibatch) and then let the inferential algorithm converge towards a fixed point. Then, we compute the mean values and the standard deviation with respect to this *M* realizations of the interaction vector $J(\tau )$. In our analysis, we choose $M=500$ different inferential procedures for subset of cardinality $n=20$.

Finally, we notice that the inferential approach followed here can also be seen as a (least squares) regression, where the regressors are all the past inter-peak durations, i.e., $z_L = \sum _{\tau } J(\tau ) z_L- \tau + \text {Gaussian noise}$. Along this line, other examples of statistical mechanics formulation include, for instance, a gaussian version of the Kinetic Ising Model (see e.g.,^40–44^).

### Generation of synthetic data

Having an estimate for the interaction vector $J(\tau )$ and for the bias *h*, we can use them to generate new synthetic sequences. From Eq. (29), we see that the conditional probability $P(z=z_{L+1}\vert \{z_n\}_{n=1}^L)$ is maximized if we take

38 $$\begin{aligned} z_{L+1}=\sum _{\tau =1}^L J(\tau )z_{L+1-\tau }+h. \end{aligned}$$

Therefore, once the first *T* components of $J(\tau )$ and the bias parameter *h* are inferred, we can generate synthetic series $\{\tilde{z}_n\}$ as follows. First, we take the first *T* points in a given experimental time-series, then we convolve it with $J(\tau )$ and add the bias, i.e.

39 $$\begin{aligned} \tilde{z}_1 = \sum _{\tau =1}^T J(\tau )z_{T+1-\tau }+h. \end{aligned}$$

Then, we can move the temporal window of length *T* along the whole experimental series in order to generate new points. Then, in general, the points in the synthetic time series are generated according to the rule

40 $$\begin{aligned} \tilde{z}_n = \sum _{\tau =1}^T J(\tau )z_{T+n-\tau }+h. \end{aligned}$$

We stress that, in our procedures, we used a stochastic version of the synthetic series generation, in which the inferred vector $J(\tau )$ is replaced with its noisy version $\bar{J}(\tau )=J(\tau )+\eta \sigma _{J(\tau )}$ with $\eta$ is a $\mathscr {N}(0,1)$ random variable. The synthetic series is then generated by collecting the $N-T$ points $\tilde{z}_n$.

### Ethical statement

Informed consent was obtained from all subjects and related data have been treated in a completely anonymous form (in the full respect of the *Declaration of Helsinki* (1964) with the understanding and the consent of the human subjects involved in the study). APH and POLISA asked for explicit approval of the study by the responsible Ethical Committee: this approval was released to APH and POLISA on June 09 2016 by the Ethical Committee of Regione Marche (APH Hospital belongs to that region) and can be supplied upon request. All the methods were carried out in strick accordance with all the relative guidelines and regulations in Italy.
